# EbfC/YbaB: A Widely Distributed Nucleoid-Associated Protein in Prokaryotes

**DOI:** 10.3390/microorganisms10101945

**Published:** 2022-09-30

**Authors:** Tamires Fernanda Vilas Boas Cordeiro, Marco Túlio Pardini Gontijo, Genesy Perez Jorge, Marcelo Brocchi

**Affiliations:** Department of Genetics, Evolution, Microbiology and Immunology, Institute of Biology, University of Campinas (UNICAMP), Rua Monteiro Lobato 255, Campinas 13083-862, Brazil

**Keywords:** nucleoid-associated protein, histone-like protein, DNA-binding, chromosome organisation, transcription, binding domains

## Abstract

Genomic compaction is an essential characteristic of living organisms. Nucleoid-associated proteins (NAPs) are a group of small proteins that play crucial roles in chromosome architecture and affect DNA replication, transcription, and recombination by imposing topological alterations in genomic DNA, thereby modulating global gene expression. EbfC/YbaB was first described as a DNA-binding protein of *Borrelia burgdorferi* that regulates the expression of surface lipoproteins with roles in virulence. Further studies indicated that this protein binds specifically and non-specifically to DNA and colocalises with nucleoids in this bacterium. The data showed that this protein binds to DNA as a homodimer, although it can form other organised structures. Crystallography analysis indicated that the protein possesses domains responsible for protein–protein interactions and forms a “tweezer” structure probably involved in DNA binding. Moreover, sequence analysis revealed conserved motifs that may be associated with dimerisation. Structural analysis also showed that the tridimensional structure of EbfC/YbaB is highly conserved within the bacterial domain. The DNA-binding activity was observed in different bacterial species, suggesting that this protein can protect DNA during stress conditions. These findings indicate that EbfC/YbaB is a broadly distributed NAP. Here, we present a review of the existing data on this NAP.

## 1. Introduction

The genome of a living organism must be compacted in an organised process to fit into the cell. Therefore, genomic compaction is an essential characteristic for all organisms. In prokaryotes, the chromosome is compacted by a combination of factors such as molecular crowding, DNA supercoiling, DNA transactions, and association with different DNA-binding proteins [1]. Nucleoid-associated proteins (NAPs), a group of low molecular weight proteins, are among the primary proteins that play an essential role in chromosome architecture [2]. NAPs can bind to DNA as monomers, dimers, or other organised structures [2]. H-NS, IHF, HU, Fis, and Dps are well-characterised NAPs of *Escherichia coli* and other species [3].

NAPs show variations in expression level and the DNA sequences they bind to, and some exhibit preferences for sequences and/or DNA topological features located along the bacterial genome. For instance, in *Salmonella enterica*, Fis is characteristically expressed during exponential growth [4,5], whereas IHF is expressed during the transition from the exponential to the stationary phase [6]. In addition, some NAPs possess bridging activities that stabilise DNA hairpins commonly observed during the exponential growth phase, whereas others wrap or bend DNA [2]. For instance, H-NS can form H-NS–DNA filaments or DNA–H-NS–DNA bridges [1]. Furthermore, the DNA-binding properties of NAPs can be modulated by factors such as protein–protein or ion interactions, which may affect the type of interaction of NAPs with DNA.

In addition to their topological effects on DNA, NAPs modulate many biological functions [2,7,8]. For example, H-NS plays an important architectural role in genome structuring and is also a transcriptional silencer for many genes. This protein binds AT-rich sequences and inhibits the expression of genes acquired by lateral gene transfer (LGT). This activity of H-NS is considered a determinant of genomic stabilisation of exogenous DNA acquired by LGT [9,10,11]. The inhibitory effect of H-NS can be disrupted by counter-silencing factors, thereby releasing many genes from the inhibitory effects of H-NS [12]. In some pathogens, this is critical for expressing virulence genes [13]. Notably, some NAPs act as classical transcription factors; they can be inhibitors or transcriptional activators. For instance, NAPs may block promoter–RNA polymerase (RNApol) binding and/or RNApol progression in transcription bubbles by bending the DNA, thereby negatively affecting transcription.

In contrast, NAPs can allow contact between enhancer and promoter sequences by bending the DNA, thereby promoting transcription. Direct contact between NAPs and RNApol has also been reported, where the former guides the latter in the transcription process [7]. However, there have been instances where differentiation between transcription factors and NAPs was not clear [14]. NAPs are also involved in other DNA transactions such as initiation of DNA replication, recombination, site-directed recombination, transposition, DNA protection, and supercoiling [1,2,3,15]. Interestingly, a link exists between some NAPs, such as Fis, and DNA supercoiling [8]. All these events affect gene expression globally [3,8,16], and many studies with model organisms demonstrated that NAPs have essential functions in regulating different physiological and pathogenic traits. Here, we have cited a few of them [2,6,17,18,19,20,21,22,23,24,25,26].

EbfC (*erp*-binding factor, chromosomal), also known as YbaB, is a less-studied and widely distributed NAP among prokaryotes [27]. EbfC was demonstrated to have significant roles in the pathogenesis of *Borrelia burgdorferi* sensu lato (referred here as *B. burgdorferi*), which causes Lyme disease [28,29,30,31,32]. The protein structures of EbfC/YbaB of *Haemophilus influenzae* [33], *E. coli* (http://www.rcsb.org/structure/1PUG, accessed on 10 June 2022), and other bacterial species have been determined. Structural analyses did not reveal a characteristic DNA-binding domain; however, experimental studies have demonstrated that these proteins bind to DNA with distinct sequence preferences [30,34]. YbaB has been suggested to have a role in DNA repair in *E. coli*, but this does not seem to be the case for *Streptomyces* [35]. Preliminary results from our laboratory indicated that YbaB of *Salmonella enterica* is not essential for pathogenesis in a mouse model of systemic infection (unpublished results).

Despite its broad distribution, the biological role of EbfC/YbaB in many prokaryotic groups is not yet understood. Therefore, considering the critical role of NAPs in prokaryotes, this review aims to describe what is known regarding EbfC/YbaB.

## 2. Operon Structure and Expression

**Figure 1:**
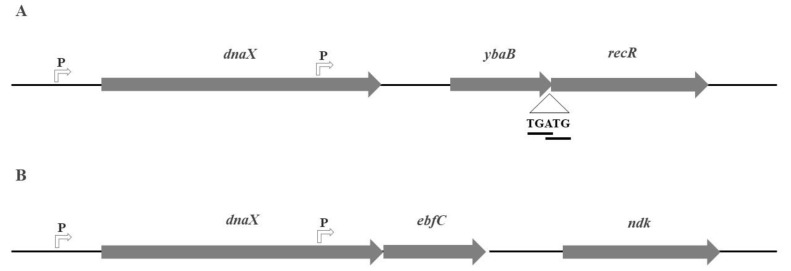
Operon structure of *dnaX*-*ybaB-recR* in *Escherichia coli* and that of *dnaX-ebfC* in *Borrelia burgdorferi*. (**A**) Operon structure of *dnaX-ybaB-recR* observed in *E. coli*. The initiation codon of *ybaB* and the termination codon of *recR* show overlap. (**B**) *dnaX-ebfC* operon of *B. burgdorferi*. The two promoters, one upstream of *dnaX* and the other located in the *dnaX* structural gene, are shown. Arrows indicate the promoter (P) and direction of transcription. The schematic structure of the operons is not to scale.

In *E. coli*, *ybaB* is located between the structural sequences of *dnaX* and *htpG* upstream of the *recR* gene. In this configuration, *ybaB* and *recR* are probably transcribed as a transcriptional unit, forming an operon [36,37]. Sequence analyses indicated the presence of an internal promoter in *dnaX*, which was shown to be a functional promoter when fused to a gene reporter [36]. Another characteristic of the *ybaB-recR* operon in *E. coli* and other bacteria is that the open reading frames of the two genes overlap, with the last “A” of the stop codon of *ybaB* corresponding to the first “A” of the start codon of *recR*. This indicates that the translation of YbaB and RecR is coupled [36,37]. Indeed, sequence analysis revealed a putative ribosome binding site (RBS) upstream of the start codon of *ybaB*, and no putative RBS was found close to the ATG of *recR*. Analyses of codon usage in *E. coli* indicated that *recR* has rarer codons than *ybaB*, which could reflect the differences in translation efficiency [36]. The operon structure of *ybaB-recR* in *E. coli* is shown in Figure 1A.

The operon structure of *ebfC* in *B. burgdorferi*, a bacterium in which the biological function of *ebfC* is best characterised, has also been described. In this bacterium, the operon *dnaX-ebfC* is regulated by a promoter located upstream of *dnaX* [30]. In addition, *ebfC* can also be expressed by a promoter located in the *dnaX* structural sequence, as determined by RT-PCR and GFP-transcriptional fusions [30,38]. In addition, DnaA, one of the main proteins of chromosomal replication, has been suggested to be involved in the regulation of expression of the *dnaX*-*ebfC* operon in *B. burgdorferi,* as discussed by Stevenson et al. [38]. The operon structure of *dnaX*-*ebfC* in *B. burgdorferi* is shown in Figure 1B.

RecR is a component of the RecFOR recombination system found in different bacterial species and is involved in DNA recombination and repair [39]. *dnaX* encodes subunits of DNA polymerase (DNApol) [40]. The organisation of *dnaX, ybaB,* and *recR* in the chromosome of *E. coli* has also been observed in other bacteria. The colocalisation of *ybaB* with *dnaX* and *recR* suggests a possible role for *ybaB* in DNA recombination, repair, and/or replication. The involvement of NAPs in the modulation of DNA transactions corroborates this suggestion. However, synteny among *dnaX-ebfC/ybaB-recR* has not been observed in many other bacterial species, which contradicts this hypothesis.

Using a two-plasmid system to identify promoters recognised by RNA polymerase containing the sigma E factor, *ybaB* belonged to the sigma E (σE) regulon in *E. coli* [41]. The σE regulon is crucial for responding to stress conditions, which could perturb cell envelope integrity [42]. Interestingly, it was demonstrated that YbaB potentiates the expression of heterologous membrane proteins in *E. coli* by an unknown mechanism [43]. This observation reinforces the possible involvement of YbaB in the envelope stress response; however, further studies are necessary to investigate their role. The biological functions assigned to *ebfC/ybaB* are discussed further in this paper.

## 3. EbfC/YbaB Orthologs Are Widely Distributed in Prokaryotes

**Figure 2:**
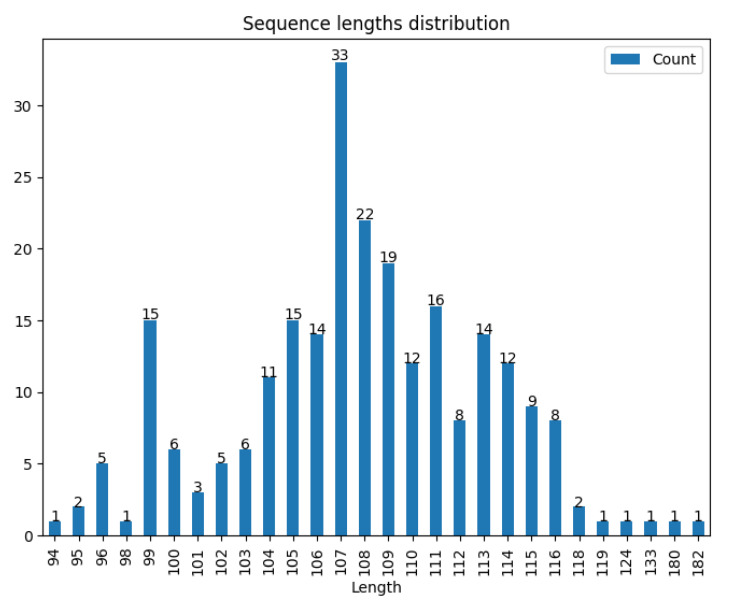
Sequence length distribution results from MetaLogo [45]. The sequences were grouped with a sequence identity cut-off of 90% using CD-HIT Suite [46], yielding 248 representative proteins. Check Supplementary Table S1 to view the accession numbers of the sequences.

In a data bank composed of 790 non-redundant, phylogenetically distinct bacterial genomes, including 704 genomes of bacteria and 86 of archaea, the most frequently found NAPs were HU, IHF, and EbfC/YbaB [27]. While this text is being written, based on the InterPro protein families and domains database [44], 585 putative reviewed sequences belong to the YbaB/EbfC family (IPR004401), and putative YbaB/EbfC was found in approximately 370 bacterial species (https://www.ebi.ac.uk/interpro/entry/InterPro/IPR004401/protein/UniProt/#table, accessed on 10 June 2022). The majority (99.19%) of putative YbaB/EbfC proteins contain 94–133 amino acids. The largest sequences comprising 180 and 182 amino acids also belonged to the YbaB/EbfC family (Figure 2). However, they are found in the genome of *Arabidopsis thaliana*, outside the bacterial domain. This might indicate that YbaB/EbfC orthologs found in bacteria might have been present in the last common ancestor of *Eukarya*, *Archaea,* and *Bacteria* or were acquired by LGT.

**Figure 3:**
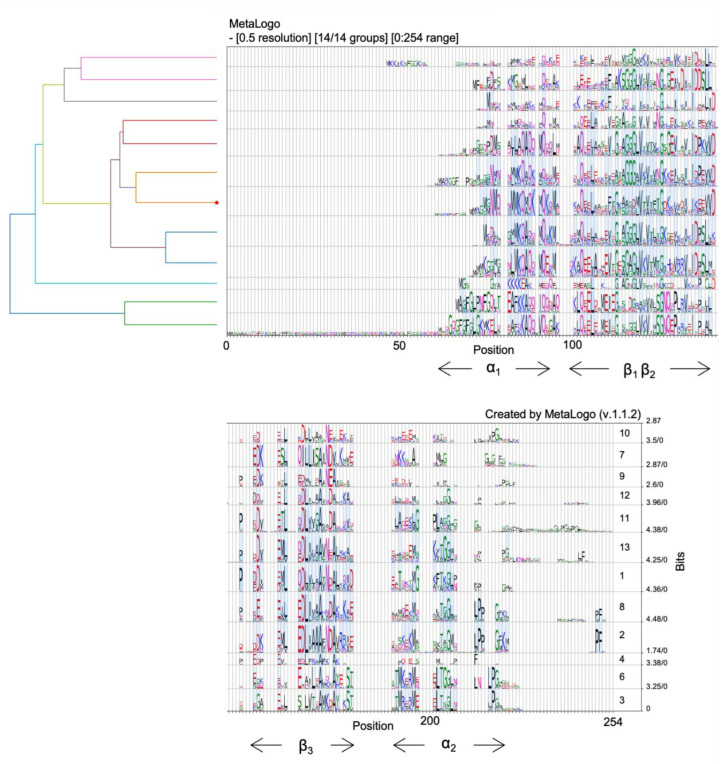
Sequence logos for all the reviewed putative proteins belonging to the YbaB/EbfC family (IPR004401). The putative sequences are deposited in the InterPro protein families and domains database [44]. The database contains 585 reviewed sequences. Sequence logos were constructed using MetaLogo [45]. The tree on the left indicates the relationships among the groups. The red dot on the tree shows the group containing the target sequence. Light blue coloured strips connect conserved positions among groups. Check Supplementary Figure S1 for a complete view of Figure 3.

Figure 3 shows the multi-group sequence logo of all 248 representative orthologs identified in the bacterial domain. The sequences were divided into 12 groups according to their evolutionary relationships, with a resolution of 0.5. Two other groups were also predicted but are not present in the cladogram due to the low number of representatives. The light blue bands highlight conserved motifs shared by adjacent groups. All regions of *ybaB* seem to be relatively conserved across bacteria (details in Section 4). However, the most conserved patterns were found to be in the β1/β2/β3 and α2 regions (also highlighted in Supplementary Figure S2B). The α1 region was also conserved in some groups, but this region appears to be less conserved than the other two. Interestingly, the α2-helix is believed to be the region directly interacting with DNA, at least in *Caulobacter crescentus* [47]. Notably, the β1/β2/β3 region is composed of amino acids with either hydrophobic side chains or small side chains. These properties may facilitate the formation of homodimers.

Supplementary Figure S2A shows the sequence counts for each group. Groups 1, 2, 8, 11, and 13 accounted for more than 70% of the total sequences. Figure S2B shows the entropy values for each position in each group. The higher the entropy (yellow), the less convergent the position. Figure S2C shows boxplots of the entropies for each group. Group 4 showed the most conserved sequence pattern with the lowest median entropies. In contrast, groups 9 and 13 showed conserved sequences. Figure S2D shows the pairwise clustering results for these groups. The data indicated four distinct clusters, one composed of homologous groups 3 and 6 (correlation coefficient > 0.75) and the other comprising groups 7, 9, and 10 (correlation coefficient > 0.50). Homologous group 4 differed the most from the others with no indicated cluster. The other homologous groups comprised the fourth cluster with a correlation coefficient greater than 0.75. However, the single Gaussian distribution pairwise distance of the sequences (Figure S2E) indicated that all homologous groups were homogeneous and must be evaluated as a whole.

## 4. EbfC/YbaB Structure

**Figure 4:**
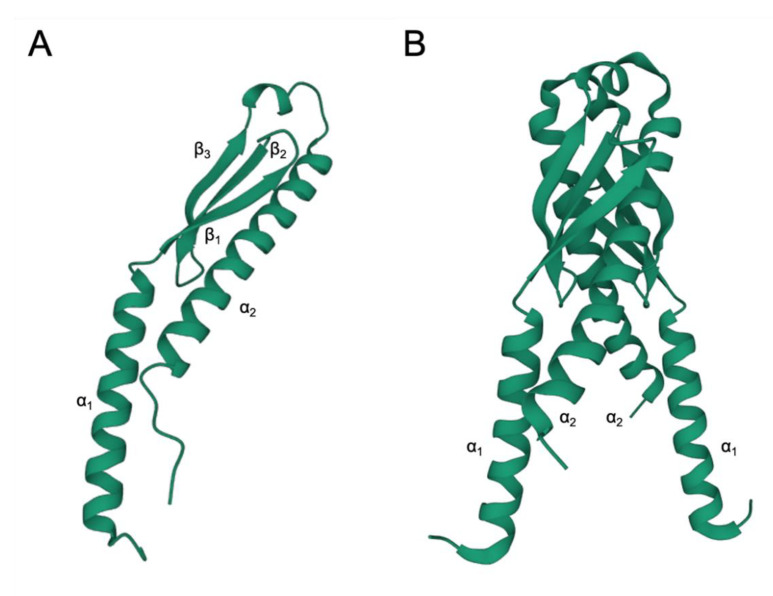
Canonical structure of EbfC/YbaB: (**A**) ribbon diagram of YbaB monomer highlighting the α + β structure with the topology N-α1/β1/β2/β3/α2-C; (**B**) ribbon diagram of the dimer, which is the most observed functional structure [33]. Structures were predicted using AlphaFold [50] and visualised using Mol*Viewer [51].

So far, the investigations have revealed that YbaB forms homodimers composed of α-helices and β-sheets with two α-helices protruding from a globular region, forming a tweezer-like structure [33,34]. To date, the crystal structure of YbaB has been determined for five organisms: *H. influenzae* (PDB accession 1j8b) [33], *E. coli* (PDB accession 1pug), *Clostridium thermocellum* (PDB accession 1ybx), *Helicobacter pylori* (PDB accession 3f42), and *Mycobacterium tuberculosis* (PDB accession 5yrx) [48]. The canonical structure of EbfC/YbaB consists of an α + β structure with the topology N-α1/β1/β2/β3/α2-C (Figure 4A). The crystals revealed a homodimer structured like a “pair of tweezers”, where the β-sheets interact, and the α-helices are extended like arms and form the putative DNA-binding domain (Figure 4B) [28,33,48]. The EbfC/YbaB protein can also form other organised structures, such as tetramers and octamers, in solution, but their properties are not apparent [29,49]. The space between the arm structures of EbfC is 15–22 Å, which would be a suitable space to fit double-stranded DNA [28].

This structure has no characteristic DNA-binding domain; however, experimental evidence showed that the EbfC (YbaB) of *B. burgdorferi*, *H. influenzae*, and *E. coli* binds to DNA [30,34]. Additionally, protein structure analyses indicated the presence of negatively charged protein-binding domains that can mimic the surface of DNA, thus permitting speculation that YbaB can compete with the DNA-binding activity of other proteins [33]. This topic is discussed in the next section.

**Figure 5:**
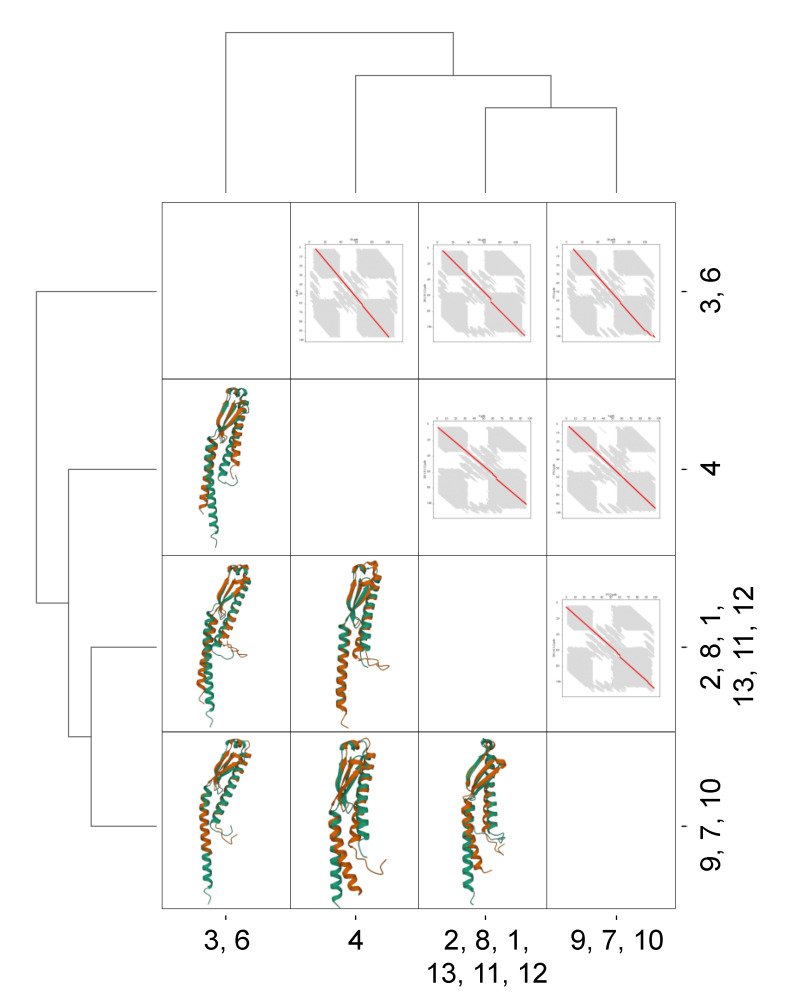
Pairwise alignment of monomeric structures of putative proteins belonging to the YbaB/EbfC family (IPR004401). Accession: Q3ANM6 (Group 3, 6), Q5L6F1 (Group 4), Q3KFA7 (Group 2, 8, 1, 13, 11, 12), and P75502 (Group 9, 7, 10). Structures were predicted using AlphaFold [50]; pairwise structural alignment was created using FATCAT [52]; and superimposed structures were visualised using Mol*Viewer [51]. The graph of the FATCAT chaining result is represented in the upper right corner, and the superimposed structures are shown in the lower left corner.

Figure 5 shows the conservation of the tridimensional structure of YbaB/EbfC across the bacterial domain. All putative proteins belonging to the YbaB/EbfC family (IPR004401) had a conserved structure (N-α1/β1/β2/β3/α2-C). Some differences were observed in the length of the α1-helix and the length of the additional amino acid chains at the N-terminus from α1 and/or the C-terminus from α2. The graph of FATCAT chaining result in Figure 5, upper right corner, revealed the significant alignment of the structures as shown by the almost straight red diagonal line, despite the small changes. Each axis represents the position of specific amino acids in the respective structure, and the red line represents their positional match compared to the pairwise structure. A straight −45° indicates a perfect pairwise alignment.

## 5. Ability to Bind to DNA

Thus far, all the data indicate that EbfC/YbaB could be grouped into a family of DNA-binding proteins. The members of this family are encoded by a broad range of bacteria [27,30,34,35,47,49,53,54,55]. Interestingly, a chloroplast-localised protein encoded by a gene (*lta1*) that controls the tiller angle and gravity response in rice and has homologs in other plants presents a conserved YbaB DNA-binding domain [56]. However, further studies are required to characterise better eukaryotic proteins with DNA-binding domains similar to EbfC/YbaB.

The DNA binding ability of EbfC/YbaB has been demonstrated in *B. burgdorferi*, *H. influenzae*, *E. coli*, *Deinococcus radiodurans*, and *C. crescentus* [28,30,34,47,55]. In *B. burgdorferi*, EbfC has been shown to bind to DNA as a homodimer, but other organised structures can also be formed [28,29]. The DNA-binding properties of *B. burgdorferi* EbfC were demonstrated using an electrophoretic mobility shift assay (EMSA). Experimental data indicated that this protein binds to DNA through its α-helical domains. As discussed above, the two α-domains form a protruding tweezer, which is hypothesised to form a DNA-binding domain. Indeed, EMSA analyses showed that EbfC protein variants carrying mutations in nine amino acids located in the α-helical regions (K16, D20, K23, N77, D78, K82, K84, E85, and K88) were unable to bind to the promoter region of *erp* genes, even at a higher concentration of mutant proteins than that of the wild-type. Protein variants were constructed by mutating wild-type amino acids with alanine [28]. These results demonstrated that the α-domains are responsible for the DNA-binding properties of EbfC. In particular, residue N77, which was predicted to be located adjacent to the β-sheet region of EbfC, was the only residue among the studied amino acids that appeared to play a role in dimerisation [28]. Interestingly, some of the residues reported to be necessary for binding EbfC to DNA (K16, D20, K23, K82, and K88) in *B. burgdorferi* [28] are not conserved in *H. influenza*. Although mutations at these residues lead to a loss of the DNA-binding capacity of the *B. burgdorferi* EbfC protein, the mutants still formed higher-order structures such as tetramers and octamers in solution, except for N77. The location of the N77 residue near the β-sheet region probably affects oligomerisation. These results indicated that EbfC multimerisation is independent of its ability to bind DNA [28].

The data obtained for the EbfC protein of *B. burgdorferi* indicated that both the N- and C-termini are involved in the formation of the α-domains and participate in DNA-binding activity [29]. To determine whether this is true for EbfC/YbaB of another bacterial species, a truncated version of the YbaB protein from *C. crescentus* lacking the putative C-terminal DNA-binding domain was constructed and tested for its binding activity to the operator sequences of *erpAB* operon. The truncated YbaB protein could not form stable protein–DNA complexes, indicating that this domain is essential for DNA-binding activity. Its removal leads to the loss of double-stranded DNA-binding activity in *C. crescentus* [47].

Available evidence suggests that EbfC/YbaB binds to DNA in sequence-specific and non-sequence-specific manners [28,30,33,34]. To date, sequence-specific binding has been described for the EbfC protein of *B. burgdorferi* [28]. EMSA, dissociation constant determination for EbfC–DNA interactions, and chromatin immunoprecipitation (ChIp) followed by sequence analysis, demonstrated that EbfC binds to the 4 bp palindromic sequence 5′-*GTnAC*-3′ with high affinity, which is spread throughout the genome of *B. burgdorferi* [28,29,30]. In addition, fluorescence microscopy analyses of *B. burgdorferi* cells containing a fusion GFP-tagged EbfC protein indicated that EbfC colocalises with bacterial nucleoids in multiple centres [30]. Further experiments demonstrated that EbfC could bind to partial or identical consensus sequences [28,29]. Although EbfC binds to the 5′-GTnAC-3′ sequence with high affinity, it can also bind to other DNA fragments lacking the palindromic sequence with lower affinity [28]. Although the YbaB orthologs of *H. influenzae* and *E. coli* exhibited preferences for specific DNA sequences, they did not show high-affinity binding for the palindromic sequence 5′-GtnAC-3′, as observed for B. burgdorferi [34]. Therefore, the preferred DNA sequences bound by YbaB in *H. influenzae* and *E. coli* are unknown, but they are probably different from those of *B. burgdorferi* [34]. Similar results were found for YbaB orthologous proteins from *D. radiodurans* and *C. crescentus* [47,55]. Both proteins were found to bind to the DNA. These proteins seem to have similar characteristics to the members of the YbaB/EbfC family, exhibiting the same tweezer-like conformation, the presence of DNA-binding domains in the N- and C-termini, and probably binding preferentially as a homodimer. The results obtained with *C. crescentus* suggested that the binding of the YbaB protein to DNA is not sequence-dependent; however, further studies are needed to clarify this question.

## 6. EbfC/YbaB Has Important Biological Functions

The functions of EbfC/YbaB have been described for several bacterial species (Table 1). For example, in *B. burgdorferi*, EbfC regulates the expression of pathogenic traits [29]. This bacterium contains multiple plasmids and independent DNA replication elements [57]. Cp32s, one of these elements, is circular and presents characteristics of a bacteriophage genome [57]. Among other traits, Cp32s contain genes that encode a polymorphic family of surface lipoproteins, the Erp proteins, involved in pathogenesis by promoting binding to plasminogen, laminin, and complement factor H, thus conferring adhesion to host tissues and inhibiting complement activation [38]. EbfC was found to regulate the transcription of *erp* operons by binding to operator sequences in the promoter region of *erp* genes. Both EbfC and BpaB (borrelial ParB) bind to the regulatory region of the *erp* operons. BpaB is a repressor that inhibits the transcription of *erp*. This protein binds cooperatively to the *erp* operator, thereby blocking the binding of RNApol to the promoter. This repression is enhanced by the binding of BpuR (borrelial PUR domain), another DNA-binding protein [38,58]. EbfC functions as an anti-repressor of *erp* operons and competes with BpaB to bind to the regulatory region, thus stimulating the transcription of *erp* genes [31,38]. *ebfC* exhibited a higher transcription level during the exponential growth phase than during the stationary phase, where transcripts were not detected. The expression of *bpaB* and *bpuR* was higher in unfed ticks in the insect’s midgut; however, *ebfC* expression was higher during the transmission of the bacteria from the feeding tick to the host, which explains the activation of *erp* transcription [30,31,38]. Therefore, the transcription of *erp* operons is inhibited by BpaB in the non-fed tick vector, but it is highly induced during the infection of mammals [30,31,38,59]. This explains why this protein is called *erp*-binding factor, chromosomal (EbfC) [28,29,30,31]. Interestingly, *bpaB* is a gene located in extra-chromosomal elements, whereas *bpuR* and *ebfC* are chromosomal genes [38].

As discussed previously, Stevenson et al. [38] recently suggested the involvement of DnaA in *dnaX*-*ebfC* expression. DnaA is necessary for DNA replication and functions as a regulatory protein [60]. However, because *dnaX* encodes DNA polymerase III subunits, these findings suggest a link between DNA replication and *ebfC* expression in *B. burgdorferi* [38].

Attempts to construct mutants of *B. burgdorferi* by *ebfC* deletion have been unsuccessful, suggesting that this is an essential protein in this species [28,31]. However, this is not the case for other species such as *E. coli*, *Streptomyces lividans*, and *Deinococcus radiodurans* [35,55,61].

As an alternative method for analysing the effects of EbfC on global gene expression, the EbfC protein was overexpressed in *B. burgdorferi*, and the transcriptome was analysed using RNA-seq [30]. Upon comparison, the abundance of *ebfC* transcripts was 28-fold in the bacteria overexpressing EbfC than in the uninduced control. This overexpression did not measurably affect the growth rate; however, transcriptome analyses indicated that EbfC affects gene expression globally in *B. burgdorferi*. In the EbfC-overexpressing strain, the expression levels of 52 genes were affected positively or negatively, representing approximately 4.5% of the gene content of the bacterial strain studied [30]. The genomic distribution of genes affected by EbfC overexpression was biased toward small replicons, an exciting observation for a NAP. However, the genomic characteristics of *B. burgdorferi* may explain this observation. The authors specified that smaller replicons carry most infection-related genes, whereas the chromosome is rich in housekeeping genes [30]. The functions of most of the genes affected by EbfC overexpression were unknown (29%), followed by metabolic processes (23%); lipoproteins (21%); DNA recombination, replication, and repair (9%); chemotaxis (8%); and others (10%) [30]. For instance, genes induced by EbfC overexpression included the genes encoding outer membrane proteins, such as *dbpB*, involved in adherence to host tissues, enzymes such as alanine racemase, and the gamma subunit of exodeoxyribonuclease V. The genes repressed by EbfC included *flhB,* which encodes a flagellar assembly/export protein, and *bbb07,* an outer membrane protein involved in tick colonisation [30].

**Table 1:** Functions of EbfC/YbaB in gene regulation and in other cellular processes as described in the literature.

Biological Roles of EbfC/YbaB	Bacterial Species	Ref.
**Regulatory functions and DNA Repair/Protection**		
Component of *sigma E* regulon (envelop stress response)	*Escherichia coli*	[41]
Regulation of pathogenic factors (EbfC as anti-repressor, stimulating the transcription of the *erp* operon)	*Borrelia burgdorferi*	[29]
Global regulator of gene expression	*Borrelia burgdorferi*	[30]
UV survival	*Deinococcus radiodurans* *Escherichia coli*	[55,61]
DNA protection from enzymatic action	*Deinococcus radiodurans* *Caulobacter crescentus*	[47,55]
DNA protection under iron-limiting conditions	*Paenibacillus riograndensis*	[62]
**Cellular processes involving EbfC/YbaB**		
Higher expression of several heterologous membrane proteins	*Escherichia coli*	[43]
EbfC/YbaB target of ClpYQ protease	*Escherichia coli*	[63]

In some bacterial species, *ebfC/ybaB* has been suggested to play a role in DNA repair by forming an operon with *recR*. Indeed, results indicate that YbaB plays a role in DNA repair because a null-mutant *E. coli* exhibited higher sensitivity to radiation than the wild-type strain [61]. However, this is not a general characteristic. *Streptomyces* is a genus of bacteria frequently used to study DNA repair because genetic instability is a common characteristic in this group. For example, a study to characterise the *recR* gene of *Streptomyces* demonstrated that mutants for *recR* or *orf1recR* of *S. lividans* presented an increased susceptibility to DNA-damaging agents [35]. Both mutant strains were complemented with the *recR* gene alone. Considering that *orf1* is orthologous to *ebfC/ybaB*, *orf1* does not seem essential for the repair function in *S. lividans* [35]. Interestingly, the same mutant strains were complemented by *orf107recR* from *Bacillus subtilis*; however, attempts to complete them with *B. subtilis recR* alone or with the *ybaBrecR* region of *E. coli* failed [35]. *orf107* is also orthologous to the *ebfC/ybaB* gene. In addition, *dr0199*, a gene orthologous to *ebfC* in *Deinococcus radiodurans*, has been suggested to play a role in DNA repair [55]. However, this activity may be due to the binding and protection of DNA, which is discussed further ahead in the text. During the construction of *ybaB* mutants, the occurrence of polar effects on *recR* should be considered. This is particularly important in species in which *ybaB* and *recR* translations are coupled, such as *E. coli*. Based on all these results, further studies are necessary to characterise better the role of EbfC/YbaB protein in DNA repair.

Studies have also indicated that EbfC/YbaB may participate in the stress response [41,55,62]. As discussed above, *ybaB* appears to belong to the σ^E^ regulon of *E. coli,* as demonstrated by a two-plasmid system [41]. However, no data indicate its participation in the *S. enterica* σ^E^ regulon [64,65], a phylogenetically related species belonging to the *Enterobacteriaceae* family. The σ^E^ regulon is activated by a cascade of events in response to misfolded and/or mis-translocated outer membrane proteins or LPS in Gram-negative bacteria as a consequence of exposure to different stress conditions [66]. In *E coli*, σ^E^ is an essential gene; however, compensatory mutations can generate variants that can survive.

In contrast, this gene is not essential in *S. enterica,* and the mutants are viable [66]. Interestingly, when co-expressed with recombinant prokaryotic or eukaryotic membrane proteins, YbaB can enhance the expression and accumulation of these proteins in *E. coli*, in some cases by a factor of 10-fold [43]. Therefore, YbaB appears to be a general “enhancer” of membrane protein production in *E. coli* [43]. These results reinforce the possible link between YbaB and the cell envelope; however, further analyses are needed to describe better their functions related to the envelope stress response.

Studies with different bacteria have suggested a possible role for EbfC/YbaB in DNA protection. *D. radiodurans* is a model organism for studying DNA repair based on its high capacity to repair DNA damage caused by mutagenic agents. This bacterium contains a gene (*dr0199*) orthologous to EbfC. A strain carrying a deletion mutation in *dr0199* exhibited high sensitivity to hydrogen peroxide and UV and gamma radiation, which was complemented by the reintroduction of the wild-type gene [55]. The authors suggested that the protective effect on DNA can be mediated by direct DNA binding; however, this protein seemed to modulate the expression of different genes in *D. radiodurans*. In *Paenibacillus riograndensis*, a plant growth-promoting bacterium, the *ebfC*/*ybaB* ortholog (0116.0006 0.0231) was highly expressed during growth in an iron-limiting medium when compared to growth under sufficient iron conditions [62]. The mechanism involved in activating 0116.0006 0.0231 expression was not determined; however, the authors suggested a function of DNA protection for this EbfC/YbaB orthologous gene [62]. Furthermore, recent characterisation of YbaB in *C. crescentus* demonstrated that this protein binds to DNA in a non-specific manner, can compact DNA, and protects it against enzymatic degradation [47]. The role of DNA-binding proteins, including some NAPs, in protecting DNA has recently been reviewed [67].

Protein turnover is an essential characteristic of any organism and is necessary for modulating peptide and protein levels, including misfolding and regulatory proteins. In *E. coli*, ClpYQ is an ATP-dependent protease, and YbaB/EbfC was demonstrated to be one of the targets of this protease [63]. These data indicated that the regulation of YbaB levels in cells is essential for *E. coli*, reinforcing the need for further studies to better characterise the roles of this NAP in prokaryotes.

## 7. Conclusions

EbfC/YbaB is a widely distributed family of prokaryote proteins capable of binding to DNA, as demonstrated in different studies. Furthermore, the structure of these proteins is conserved, exhibiting a general topology of N-α1/β1/β2/β3/α2-C, with regions involved in dimerisation and DNA-binding. The DNA-binding region, structured as a pair of tweezers, probably represents a DNA-binding domain that is not well-characterised yet. Despite its wide distribution, the roles of EbfC/YbaB have been described in a few bacterial species. In *B. burgdorferi*, EbfC binds specifically to DNA sequences with high affinity. However, this protein can also bind to DNA sequences in a lower affinity non-specific manner. Data from the literature suggest that EbfC/YbaB orthologs may be involved in the bacterial envelope stress response, DNA protection, and repair. However, further studies are required to clarify the role of EbfC/YbaB orthologs in these processes. EbfC of *B. burgdorferi* is undoubtedly the best-characterised EbfC/YbaB ortholog in terms of DNA binding, regulation of pathogenic traits, and as a global regulator. Taken together, these data suggest that EbfC/YbaB is a NAP, but its biological function has not yet been characterised in most prokaryotic species. Therefore, further studies are necessary to better describe the biological roles of this NAP in prokaryotes.

## Data Availability

Not applicable.

## Supplementary Materials

The following supporting information can be downloaded at: https://www.mdpi.com/article/10.3390/microorganisms10101945/s1, **Figure S1:** Sequence logos for all the reviewed putative proteins belonging to the YbaB/EbfC family (IPR004401). The putative sequences are deposited in the InterPro protein families and domains database [1]. The database contains 585 reviewed sequences. Sequence logos were constructed using MetaLogo [2]. The tree on the left indicates the relationships among the groups. The red dot on the tree indicates the group containing the target sequence. Light blue coloured strips connect conserved positions among groups; **Figure S2**: Results obtained from MetaLogo [2]. (**a**) Sequence counts of each group; (**b**) Entropy heatmap of each group; (**c**) Boxplot of entropies of positions in all groups; (**d**) Clustering result of sequence logo groups revealing the pairwise relationships; (**e**) Distribution of pairwise distances of the nodes in the phylogenetic tree; **Table S1:** Homologous group, accession to InterPro database, strain ID, sequence length, and sequence match for YbaB/EbfC.
