# Supplementary material for: EbfC/YbaB: A Widely Distributed Nucleoid-Associated Protein in Prokaryotes
Source: Microorganisms. 2022 Sep 30;10(10):1945. doi: 10.3390/microorganisms10101945 (PMC9610160; doi:10.3390/microorganisms10101945)
Supplement: Supplementary file 1 [file microorganisms-10-01945-s001.zip › Supplementary Figures.pdf]

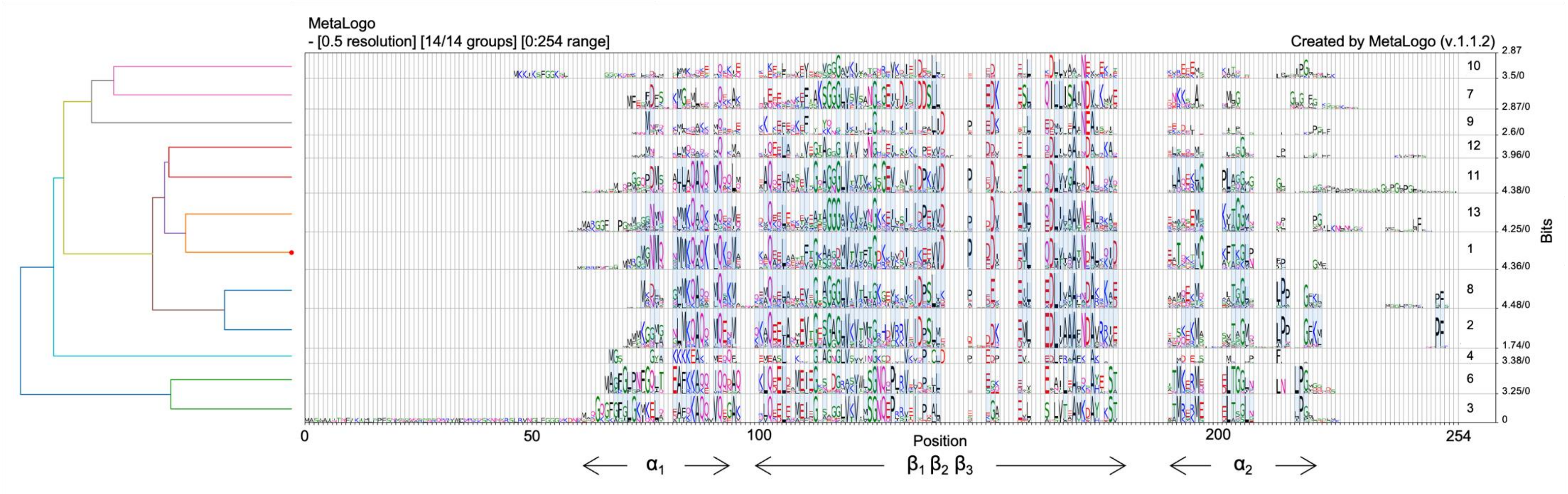

Supplementary Figure S1. Sequence logos for all the reviewed putative proteins belonging to the YbaB/EbfC family (IPR004401). The putative sequences are deposited in the InterPro protein families and domains database [1]. The database contains 585 reviewed sequences. Sequence logos were constructed using MetaLogo [2]. The tree on the left indicates the relationships among the groups. The red dot on the tree indicates the group containing the target sequence. Light blue coloured strips connect conserved positions among groups.

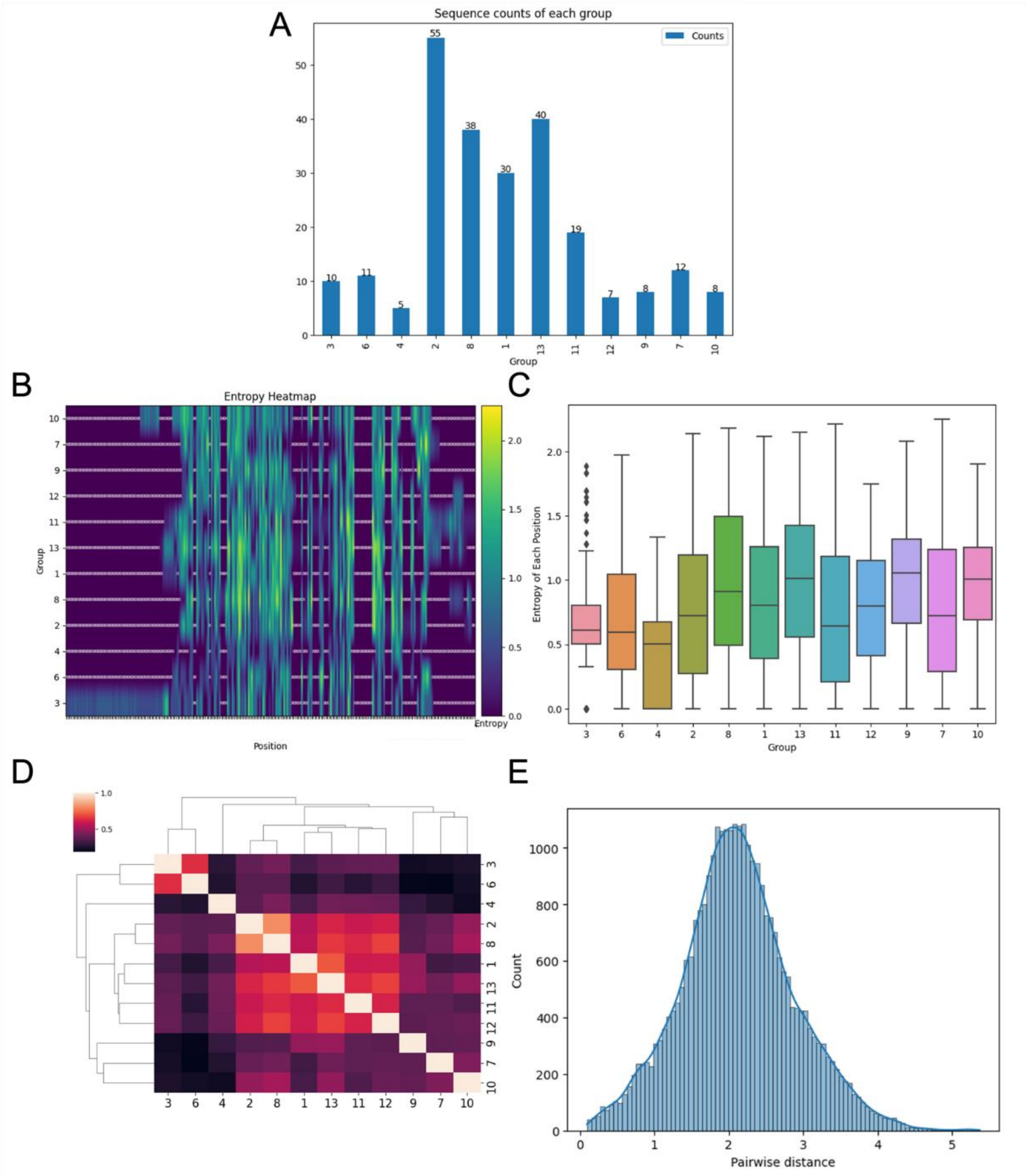

Supplementary Figure S2. Results obtained from MetaLogo [2]. (**a**) Sequence counts of each group; (**b**) Entropy heatmap of each group; (**c**) Boxplot of entropies of positions in all groups; (**d**) Clustering result of sequence logo groups revealing the pairwise relationships; (**e**) Distribution of pairwise distances of the nodes in the phylogenetic tree.

References

1. Blum, M.; Chang, H.-Y.; Chuguransky, S.; Grego, T.; Kandasaamy, S.; Mitchell, A.; Nuka, G.; Paysan-Lafosse, T.; Qureshi, M.; Raj, S.; et al. The InterPro Protein Families and Domains Database: 20 Years On. *Nucleic Acids Res* **2021**, *49*, D344–D354, doi:10.1093/nar/gkaa977.
2. Chen, Y.; He, Z.; Men, Y.; Dong, G.; Hu, S.; Ying, X. MetaLogo: A Heterogeneity-Aware Sequence Logo Generator and Aligner. *Brief Bioinform* **2022**, *23*, bbab591, doi:10.1093/bib/bbab591.
